# Influence of Tempo and Rhythmic Unit in Musical Emotion Regulation

**DOI:** 10.3389/fncom.2016.00080

**Published:** 2016-08-03

**Authors:** Alicia Fernández-Sotos, Antonio Fernández-Caballero, José M. Latorre

**Affiliations:** ^1^Facultad de Educación de Albacete, Universidad de Castilla-La ManchaAlbacete, Spain; ^2^Departamento de Sistemas Informáticos, Instituto de Investigación en Informática de Albacete, Universidad de Castilla-La ManchaAlbacete, Spain; ^3^Facultad de Medicina de Albacete, Universidad de Castilla-La ManchaAlbacete, Spain

**Keywords:** emotion regulation, music, note value, tempo, rhythmic unit

## Abstract

This article is based on the assumption of musical power to change the listener's mood. The paper studies the outcome of two experiments on the regulation of emotional states in a series of participants who listen to different auditions. The present research focuses on note value, an important musical cue related to rhythm. The influence of two concepts linked to note value is analyzed separately and discussed together. The two musical cues under investigation are tempo and rhythmic unit. The participants are asked to label music fragments by using opposite meaningful words belonging to four semantic scales, namely “Tension” (ranging from *Relaxing* to *Stressing*), “Expressiveness” (*Expressionless* to *Expressive*), “Amusement” (*Boring* to *Amusing*) and “Attractiveness” (*Pleasant* to *Unpleasant*). The participants also have to indicate how much they feel certain basic emotions while listening to each music excerpt. The rated emotions are “Happiness,” “Surprise,” and “Sadness.” This study makes it possible to draw some interesting conclusions about the associations between note value and emotions.

## 1. Introduction

Brain structures and networks related to music processing of many kinds, including music perception, emotion and music, and sensory processing and music, have been discovered by psychologists and cognitive neuroscientists (Hunt, 2015). Recently, there has been an increase of research that relates music and emotion within neuropsychology (see e.g., Peretz, 2010). Many studies in neuropsychology and music have investigated reactions to specific musical cues (Koelsch and Siebel, 2005), such as melody (Brattico, 2006), harmony including basic dissonance-consonance (Koelsch et al., 2006), modality in terms of major-minor (Mizuno and Sugishita, 2007), rhythm (Samson and Ehrlé, 2003), and musical timbre (Caclin et al., 2006).

This article follows the findings on the assumption of the power of music to regulate the listeners mood (Fernández-Sotos et al., 2015). Indeed, emotion regulation through music is often considered one of the most important functions of music (Saarikallio and Erkkila, 2007). The two experiments presented in this paper are part of a project named “Improvement of the Elderly Quality of Life and Care through Smart Emotion Regulation” (Castillo et al., 2014b, 2016; Fernández-Caballero et al., 2014). The project's general objective is to find solutions for improving the quality of life and care of aging people who wish to continue living at home with the aid of emotion elicitation techniques. Cameras and body sensors are used for monitoring the aging adults' facial and gestural expression (Lozano-Monasor et al., 2014), activity and behavior (Castillo et al., 2014a), as well as for acquiring relevant physiological data (Costa et al., 2012; Fernández-Caballero et al., 2012; Martínez-Rodrigo et al., 2016). By using advanced monitoring techniques, the older people's emotions should be inferred and recognized. On the other hand, music, color and light are the proposed stimulating means to regulate their emotions toward a “positive” mood in accordance with the guidelines of a physician.

This paper introduces an initial step of the project that focuses on specific musical cues related to note value. Note value is the duration of a note, or the relationship of a note's duration to the measure. To sum up, the current paper introduces some hints in the projects overall aim to investigate the listeners changes in emotional state through playing different auditions that have been composed under defined parameters of note value. This way, it will be possible to conclude if and how the analyzed musical cues are able to induce positive and negative emotions in the listener.

In this sense, the article describes a couple of experiments that are aimed at detecting the individual influential issues related to two basic components of note value, that is, tempo and rhythmic unit. Tempo (time in Italian) is defined as the speed of a composition's rhythm, and it is measured according to beats per minute. Beat is the regular pulse of music which may be dictated by the rise or fall of the hand or baton of the conductor, by a metronome, or by the accents in music. On the other hand, a rhythmic unit is defined as a durational pattern that synchronizes with a pulse or pulses on the underlying metric level.

Tempo and rhythm have been studied in many previous works as broad concepts. As far as we know, it is the first time that tempo and rhythmic unit are studied as intrinsic parameters of note vale. In this paper, the individual influence of tempo and rhythmic unit are experimentally studied. In addition, from the results of both experiments carried out, the two parameters are related in their emotional influence. Lastly, this article established a basis for future study of the combined effect of both parameters.

## 2. Related work

It has been well established that arousal and mood represent different but related aspects of emotional responding (Husain et al., 2002). Although the use of these terms in the literature varies, mood typically refers to relatively long lasting emotions (Sloboda and Juslin, 2001), which may have stronger consequences for cognition than action. Arousal typically refers to the degree of physiological activation or to the intensity of an emotional response (Sloboda and Juslin, 2001). According to the arousal-mood hypothesis, listening to music affects arousal and mood, which then influence performance on various cognitive skills. The impact of music on arousal and mood is well established. People often choose to listen music for this very effect (Gabrielsson, 2001), and physiological responses to music differ depending on the type of music heard.

A research work on the impact of individual musical cues in the communication of certain emotions to the listener states that the most potent and frequently studied musical cues are mode, tempo, dynamics, articulation, timbre, and phrasing (Gabrielsson and Lindstrom, 2010). In fact, musical parameters such as tempo or mode are inherent properties of musical structure (van der Zwaag et al., 2011), which is known to influence listeners emotions. Music preferences commonly are treated as affective states (Scherer and Zentner, 2001), as they are strongly linked to valence (positive or negative experiences) (Istók, 2013). Moreover, recognizing basic emotions in music such as happiness or sadness is effortless and highly consistent among adults (Peretz et al., 1998).

In an example of experimentation with musical cues, twenty performers were asked to manipulate values of seven musical variables simultaneously (tempo, sound level, articulation, phrasing, register, timbre, and attack speed) for communicating five different emotional expressions (neutral, happy, scary, peaceful, sad) for each of four scores (Bresin and Friberg, 2011). Also, another research has revealed that there are a number of elements in sound aspects that modify emotional responses when listening to music (Glowinski and Camurri, 2012). These elements are connected to score features such as pitch (high/low), intervals (short/long), harmony (consonant/dissonant) and rhythm (regular/irregular). The dynamic between these sound elements is also important and depends largely on instrumental interpretation (or performance features), e.g., rhythmic accents, articulation (staccato/legato), variations in timbre (spectral richness, playing mode, and so on). In a systematic manipulation of musical cues, an optimized factorial design was used with six primary musical cues (mode, tempo, dynamics, articulation, timbre, and register) across four different music examples (Eerola et al., 2013). In the last 10 years, many other studies have assessed the influence of tempo, mostly combined with mode, in affective reactions. An interactive approach to understanding emotional responses to music was undertaken by simultaneously manipulating three musical elements: mode, texture, and tempo (Webster and Weir, 2005).

In our understanding all these previous studies show a need for going on in experimenting with tempo and rhythmic unit for the sake of regulating emotions through music.

## 3. Materials and methods

### 3.1. Description of the experimentation

Firstly, we have to highlight that our aim is to investigate solely the influence of note value parameters on emotional reactions. This is why all the musical fragments used in the two experiments have been designed in major mode. The results offered in this article would probably not apply in minor mode musical pieces in line with a series of studies (e.g., Husain et al., 2002; Knoferle et al., 2012). In Husain et al. (2002), the effects of tempo and mode in spatial abilities, levels of arousal and mood are discussed. Thus, listeners are offered four musical versions in which tempo (fast and slow) and mode (major and minor) are varied, so that they can judge them after listening. It is concluded that the performance of a spatial task increases with increasing tempo when listening in major mode. By contrast, the performance decreases with listening in slower tempo and minor mode. Similarly, in Knoferle et al. (2012) there is an experiment on the effects of mode and tempo in improving sales (marketing). During 4 weeks, play lists were placed in different shops under the same conditions (330 pop and rock songs with variations in tempo between 95 and 135 beats per minute (bpm), in major and minor mode, divided into four sets of songs, major-rapid, minor-fast, major-slow and minor-slow) for the sake of analyzing the increase or not in shopping. Thus, it is concluded that listening to music in a major mode and fast tempo is much more effective than listening to music in minor mode and slow tempo. Both studies suggest the existence of an optimal tempo for major and minor mode.

Sixty three young people (males and females) aged between 19 and 29 years have participated in the two experiments. Participants are students of the subject “Musical Perception and Expression,” taught by the first author of this paper. The students are enrolled in the 3rd year course of an Early Childhood Education Degree offered at Albacete School of Education, Spain. Some of these students are willing to teach music to preschool children in a close future. This study was carried out in accordance with the ethical standards and with the approval of the Ethics Committee for Clinical Research of the University Hospital Complex of Albacete (Spain) with written informed consent from all subjects. All subjects gave written informed consent in accordance with the Declaration of Helsinki.

The experimentation is carried out in a specially organized room, where each participant is placed in front of a computer. The evaluation of musical influence is performed with a software application. Each participant is asked to judge about each of the music pieces played on the computer at a volume of 20 decibels (dB). The participants are asked to label the music pieces for the following indirect semantic scales, where each scale is represented by two opposite descriptive words:

- “Tension”: from *Relaxing* to *Stressing*
- “Expressiveness”: from *Expressionless* to *Expressive*
- “Amusement”: from *Boring* to *Amusing*
- “Attractiveness”: from *Pleasant* to *Unpleasant*

**Figure 1:**
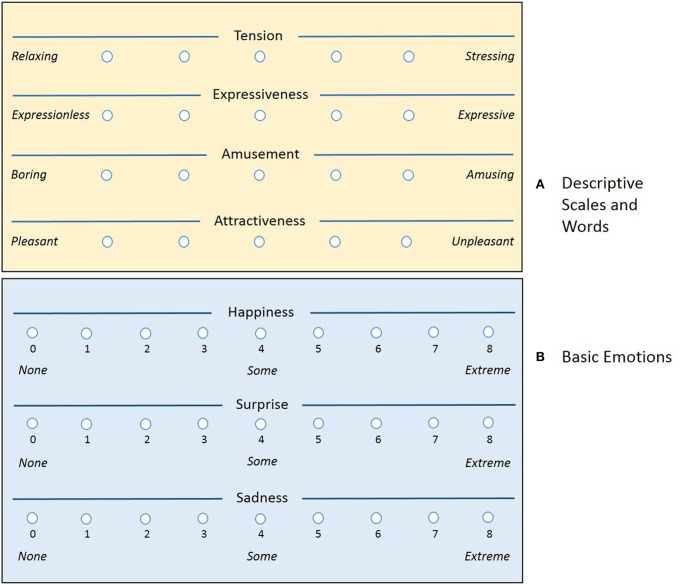
**Graphical user interface with questions**. **(A)** Descriptive scales and words. **(B)** Basic emotions.

Next, each participant reports how he/she felt one of basic emotions “Happiness,” “Surprise,” and “Sadness.” The choice of these emotions is based on the premise that the practical purpose of this study is to find musical cues that improve mood. The participants indicate a value corresponding to the intensity of each description and emotion. Each of the semantic scales is evaluated with a value ranging from 1 to 5. The self-reported emotions are also ranked similarly with a discrete integer value, now ranging from 0 to 8 (see Figure 1). As depicted in the figure, a value of 0 corresponds to *None* and 8 to *Extreme*.

### 3.2. Statistical analysis

The statistical analysis was conducted using SPSS 20.0. In our study, we have calculated the mean (M) and the standard deviation (SD) of each input parameter (related to tempo and rhythmic unit) in relation to each output (descriptive scales and basic emotions). We have calculated the percent changes in the two experiments, subtracting the value from one level to the previous and dividing it by the first, in order to make data related to variations that occur in the different emotional parameters more understandable.

An ANOVA for repeated measures was used to evaluate the effects of both tempo and rhythmic unit on descriptive scales “Tension,” “Expressiveness,” “Amusement,” and “Attractiveness,” and emotions “Happiness,” “Surprise,” and “Sadness.” In the one-factor repeated measures model, we assume that the variances of the variables are equal. This assumption is equivalent to saying that the variance-covariance matrix is circular or spherical. To test this assumption, we used the Mauchly sphericity test (W). When the critical level associated with statistic W is greater than 0.05, we cannot reject the sphericity hypothesis. In such cases, we use multivariate statistics because they are not affected by failure of the sphericity. In the *post-hoc* comparisons, critical levels are adjusted by Bonferroni correction to control the error rates or probability from making mistakes of type I. For the significance, we have considered a critical value of *p*-value < 0.05. The interpretation of η^2^ is the common one: 0.02 small; 0.13 medium; 0.26 large.

### 3.3. Musical experiment #1: influence of tempo

There is no doubt that tempo is an essential element of note value. Indeed, rhythm is based around the tempo. The tempo enables perceiving music in an organized manner. It forms the basis on which the melodic-harmonic lines are built. The promotion in children of perception, acquisition and reproduction of the tempo is a widely advocated topic. This practice has a positive effect on reading assignments, learning vocabulary, maths and motor coordination of the younger (Weikart, 2003). Children perceive better the responses that they receive from the exterior through a constant beat, allowing giving logical sense to their world. This element is present in daily actions as observed in speech and body movements made by the human being (Norris, 2009). On the one hand, there is a social synchrony between human movements; the tempo is an underlying social interaction organizer (Scollon, 1982). On this basis, Norris (2009) shows that two individuals in contact tend to synchronize their movements and they reach to establish a common beat pattern. Tempos are also observed in verbal discourse, for example when a question is posed and an answer is provided. This fact is noted in the gestures and movements associated with the discourse. Moreover, this situation also occurs in listening to background music. It is worth highlighting that the listener synchronizes his/her movements with the tempo perceived in music.

**Figure 2:**
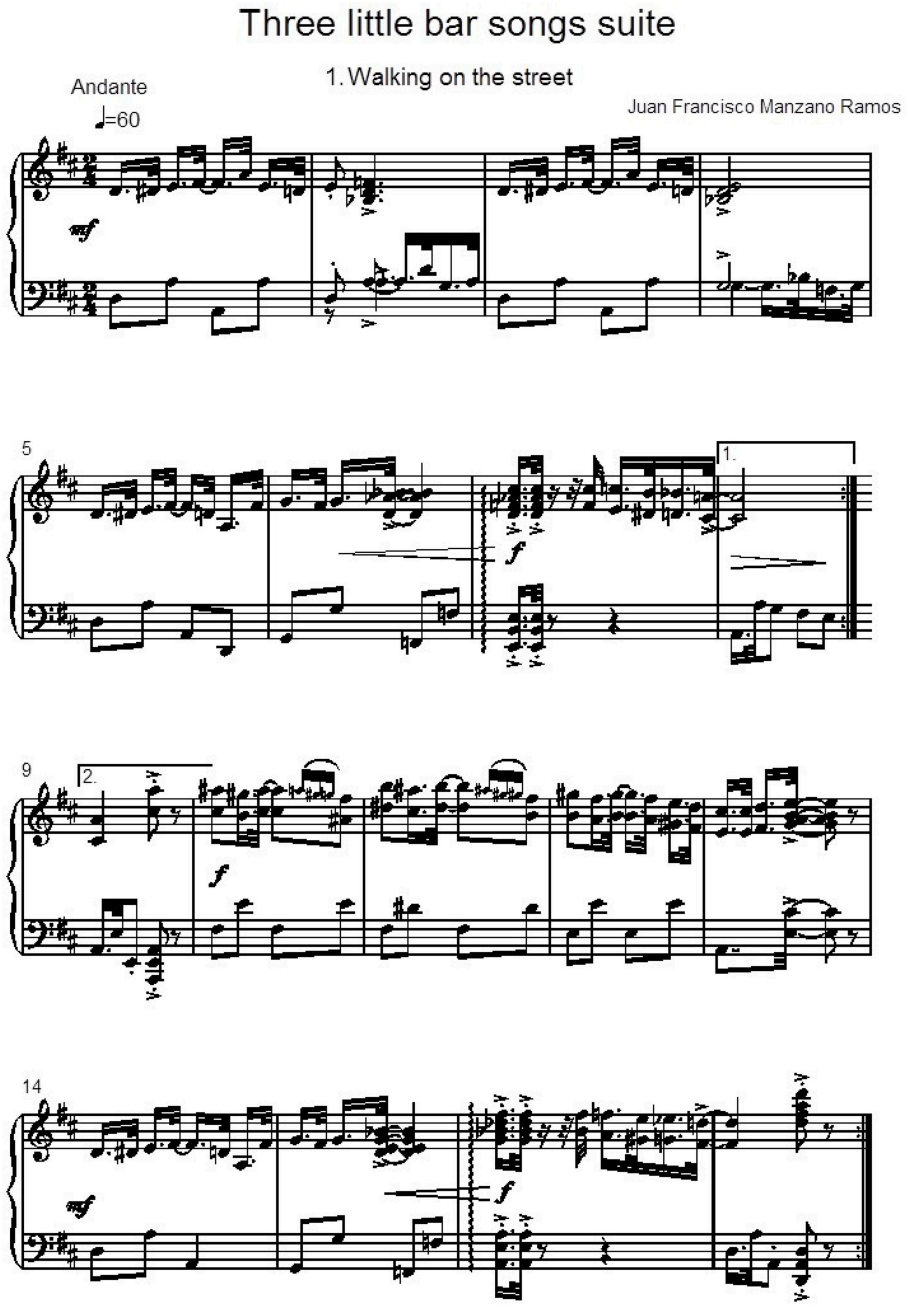
**“Walking on the Street” theme and 3 variations**.

Thus, the first musical test that is proposed here focuses on the evaluation of three tunes by the listener. The three melodies are really the same one, but it is varied on two occasions by altering the tempo. The piece is titled “Walking on the Street,” framed in a suite called “Three Little Bar Songs Suite” (see Figure 2). It has been written by the contemporary composer Juan Francisco Manzano Ramos. We wanted to start with this little piece in non-classical style to bring enough variety to the experimentation. The different musical pieces combine both classical and contemporary elements of music. The only requirement is that all musical pieces share a tonal harmonic language, with a harmonic rhythm of classical music and repetitive rhythmic parameters. This enables to highlight each of the auditions in order to categorize them correctly. So, in this way, we have a musical piece which rhythm uses constantly alternating dotted notes (providing a touch of swing) and syncopated notes in prominent places. Then, tempo variations are provided to the rhythm used.

As described before, the tempo is measured according to beats per minute. A very fast tempo, prestissimo, has between 200 and 208 beats per minute, presto has 168 to 200 beats per minute, allegro has between 120 and 168 bpm, moderato has 108 to 120 beats per minute, andante has 76 to 108, adagio has 66 to 76, larghetto has 60 to 66, and largo, the slowest tempo, has 40 to 60. In our experiment, we have decided to use only three different tempos. Tempos to be heard are 90, 120, and 150 bpm, respectively, covering a sufficient range of standard beats. The order of appearance of each melody is in increasing number of bpms for each listener in the computer program. The listener labels melodies in the way he/she considers more suited as described before. That is, he/she offers a value from 1 to 5 to each of the description-related scales, and from 0 to 8 to the basic emotions.

#### 3.3.1. Musical experiment #2: influence of rhythmic unit

In relation to the rhythm, typically the basic rhythmic patterns are addressed in duple meter, or triple meter present in the continuous movements of the human being as, for instance, walking. Let us remind that each of the categories of meter is defined by the subdivision of beats. The number of beats per measure determine the term associated with that meter. For instance, duple meter is a rhythmic pattern with the measure being divisible by two. This includes simple double rhythm such as 2/2, 4/4, but also such compound rhythms as 6/8. And, triple meter is a metrical pattern having three beats to a measure.

Jaques-Dalcroze emphasizes the importance of implementing the rhythmic movements, perceived in music and represented through the human body in its rhythmic part, in the right balance of the nervous system. Jaques-Dalcroze (1921, 1931) stresses that rhythm is movement and all motion is material. Therefore, any movement is in need of space and time. Thus, Jaques-Dalcroze starts from the binary rhythm in his teaching, associating it to freely walking. For this reason, one of the basic methodologies used is the association of half notes (basic beat in meter two by four) with walking, eighth notes with running, and eighth notes with dotted notes and sixteenth notes with jumping. Let us remind that the duration of a note is as shown in Table 1 in common time or 4/4 time.

**Table 1:** **Description of note values and associated terminology**.

**Note values**	**British term**	**American term**
Note having a duration of one full measure	Semibreve	Whole note
Note having a duration of one half of a full measure	Minim	Half note
Note having a duration of a quarter of a full measure	Crotchet	Quarter note
Note having a duration of one eighth of a full measure	Quaver	Eighth note
Note having a duration of one sixteenth of a full measure	Semiquaver	Sixteenth note
Note having a duration of one thirty-second of a full measure	Demisemiquaver	Thirty-second note

**Figure 3:**
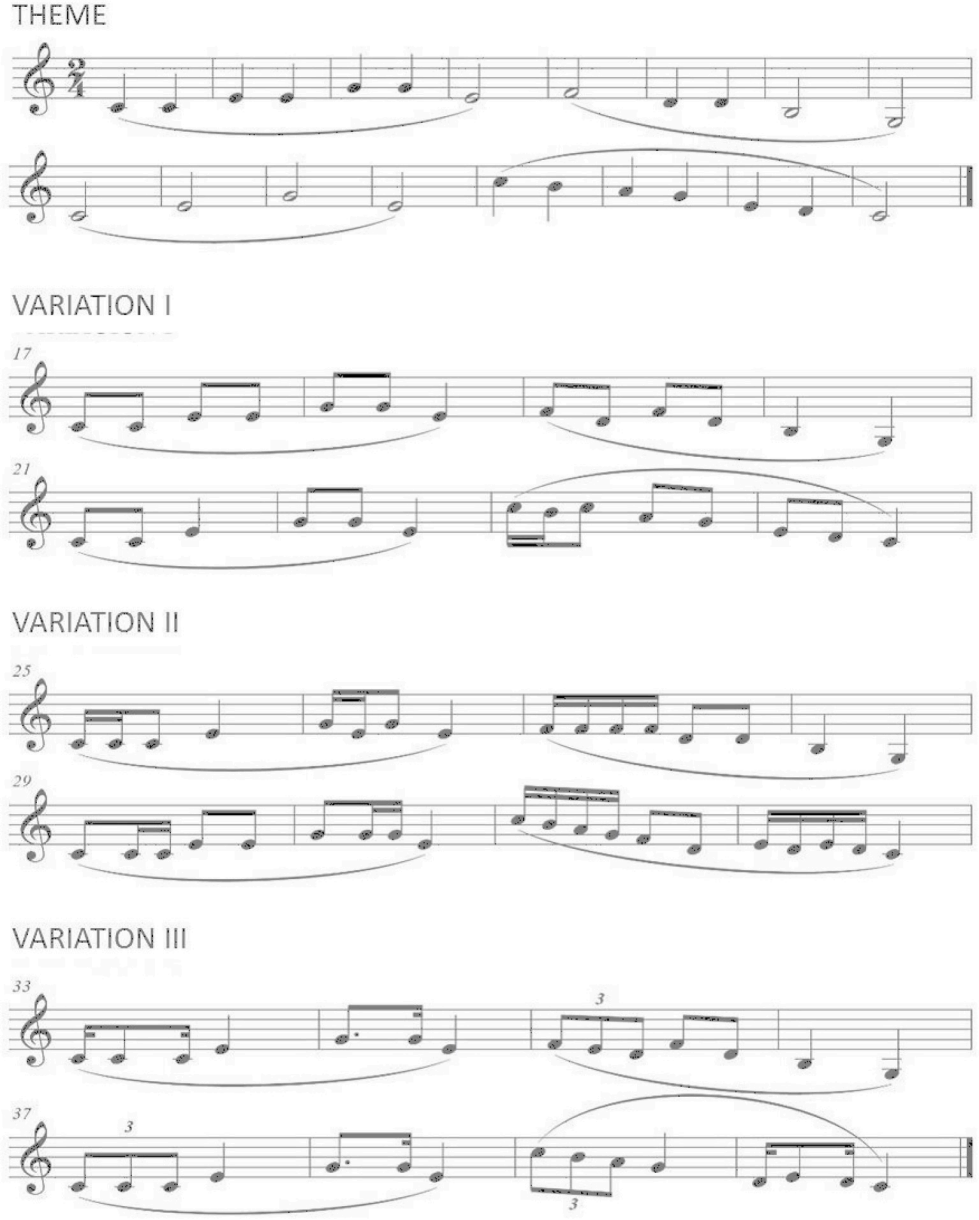
**“Surprise” theme and 3 variations**.

In this sense, the second musical experiment is geared to the variation of the rhythmic unit. Rhythmic units may be classified as metric-even patterns, such as steady eighth notes, or pulses-intrametric-confirming patterns, such as dotted eighth-sixteenth note and swing patterns-contrametric-non-confirming, or syncopated patterns and extrametric-irregular patterns, such as tuplets (also called irregular rhythms or abnormal divisions). In other words, it is the variation of the rhythm of the melody without altering the musical line, harmonics or beat. To do this, from the main melody of the symphony “Surprise” by Haydn, three rhythmic variations are established (see Figure 3). The listener hears and newly labels what the melody suggests to him/her from the list of opposing descriptive words and direct basic emotion shown on the computer interface. The principal theme is characterized by the use of rhythmic whole and half notes. Afterwards, the original melody is slightly modified, especially in the last four bars where a cadence amendment is performed. Variation 1 is characterized by the predominance of the rhythmic formula of two eighth notes. A slight variation is introduced in the sixth bar in order to bring interest to the resolution of the theme. Variation 2 is characteristic of the use of simple combinations of the representation of sixteenth notes, that is, rhythmic formulas of four sixteenth notes, two sixteenth-eighth notes and eighth-two sixteenth notes. Finally, variation 3 uses syncopated notes, dotted notes and triplets, all in value of a beat.

## 4. Results

The results of both experiments are described separately for a better understanding of the singular influence of the tempo and the rhythmic unit on the listener's emotional state.

### 4.1. Results of musical experiment #1: influence of tempo

Table 2 shows in columns 2 to 4 the means and standard deviations for the three tempos (90, 120, and 150 bpm) used during the experimentation.

**Table 2:** **Descriptive statistics and ANOVA test for experiment 1: The tempo**.

**Tempo (*n* = 63)**	**90 bpm *M* (*SD*)**	**120 bpm *M* (*SD*)**	**150 bpm *M* (*SD*)**	***F* (DF) (1.62)**	**Sig. *p***	**η^2^**	**A vs. B *p***	**A vs. C *p***	**B vs. C *p***
**(Range 1–5)**									
Tension	2.27 (0.95)	2.63 (0.84)	3.16 (0.98)	28.00	0.000	0.311	0.016	0.000	0.000
Expressiveness	3.79 (0.98)	4.00 (0.69)	4.29 (0.70)	16.96	0.000	0.215	0.140	0.000	0.006
Amusement	3.43 (0.99)	3.98 (0.70)	4.24 (0.79)	37.80	0.000	0.379	0.000	0.000	0.051
Attractiveness	2.57 (1.07)	2.52 (1.03)	2.46 (1.10)	0.55	0.457	0.009	1.000	1.000	1.000
**(Range 0–8)**									
Happiness	4.35 (1.80)	4.97 (1.69)	5.65 (1.47)	41.54	0.000	0.401	0.003	0.000	0.001
Sadness	1.30 (1.07)	0.81 (0.98)	0.49 (0.73)	42.86	0.000	0.409	0.006	0.000	0.006
Surprise	3.62 (1.93)	4.05 (1.97)	4.17 (1.93)	5.59	0.021	0.083	0.107	0.064	1.000

Multiple comparisons a posteriori using Bonferroni test: 90 bpm (A), 120 bpm (B) and 150 bpm (C). Critical value of p < 0.050.

With the augmentation of tempo (from 90 to 150 bpm), there is an increase in the mean values of emotions “Happiness” and “Surprise.” There is a similar behavior in semantic scales “Tension,” “Expressiveness,” and “Amusement.” In addition, there is a decrease in the mean values of “Sadness” emotion. In relation to Attractiveness” scale, we have to conclude that there is no significant change. The mean values are similar for all three tempos. From this point on, we will no longer study the results of “Attractiveness” provided in this experiment number 1.

**Figure 4:**
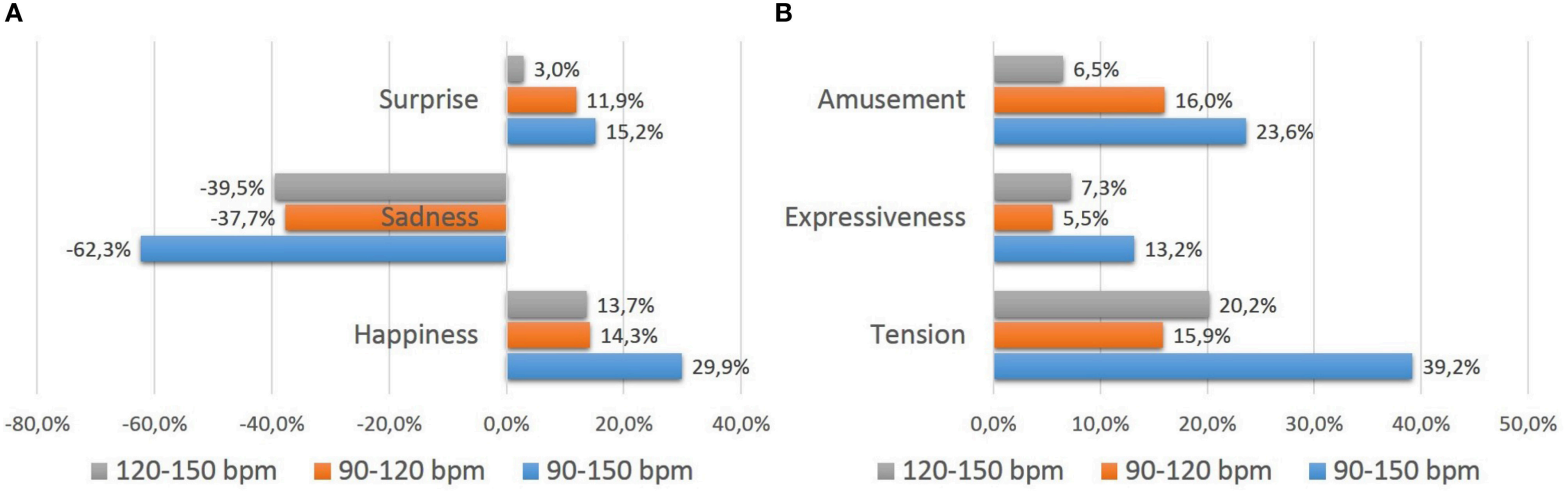
**Growth tendencies for experiment 1**. **(A)** Tendencies in basic emotions. **(B)** Tendencies in descriptive scales.

Moreover, there are significant differences in growth percentages for the parameters when studying the increase/decrease from 90 to 150 bpm (see Figure 4). Indeed, there are emotional factors that suffer large variations, while variation is not so relevant in others. The emotional perception of “Sadness” is the most affected with increasing tempo. The other emotions are less affected. In relation to descriptive scales, all of them experiment and increase, following the next order: “Tension,” “Amusement,” and “Expressiveness.”

It seems also useful to investigate the partial variations due to the augmentation from 90 to 120 bpm and from 120 to 150 bpm. Let us start with the first augmentation. The perception of emotion “Sadness” decreases by 37.7%, which the highest percentage for all emotions and descriptive words. Other four parameters increase their values in a margin from around 12 to 16% when augmenting the pulse from 90 bpm to 120 bpm. The scores of “Amusement” and “Tension” scales increase by 16.0 and 15.9%, while valuations of emotions “Happiness” and “Surprise” rise by 14.3 and 11.9%, respectively. The last parameter studied, namely “Expressiveness,” does not experience much variation in the results of emotional perception when the initial pulse is increased by 30 bpm. The score of “Expressiveness” only increases by 5.3%.

Moreover, when the tempo is augmented again (from 120 to 150 bpm), very different growth patterns are observed between the emotions and descriptive scales studied. In line with our previous assertion it is seen that the values of emotions “Happiness,” “Surprise,” and the scales “Amusement,” “Expressiveness” and “Tension” continue increasing. Again, the punctuation of emotion “Sadness” descends by 39.5%. Very different behaviors are observed here. Newly, among the descriptive words that do increase their values, only one of them stands out. Indeed, “Tension” is the only term that increases its growth with this tempo acceleration by 4.3% higher than the 15.9% previously experienced.

### 4.2. Results of musical experiment #2: influence of rhythmic unit

Table 3 has a very similar layout to Table 2. Here, columns 2 to 5 offer the measures and standard deviations for the theme and three variations performed in this experiment.

**Table 3:** **Descriptive statistics and ANOVA test for experiment 2: The rhythmic unit**.

**Rhythmic unit (*n* = 63)**	**Theme *M* (*SD*)**	**Variation 1 *M* (*SD*)**	**Variation 2 *M* (*SD*)**	**Variation 3 *M* (*SD*)**	***F* (DF) (1.62)**	**Sig. *p***	**η^2^**	**A vs. B *p***	**A vs. C *p***	**B vs. C *p***
**(Range 1–5)**										
Tension	2.03 (1.17)	2.76 (0.83)	3.13 (0.95)	2.98 (1.07)	23.34	0.000	0.274	0.000	0.000	0.028
Expressiveness	2.19 (0.73)	3.37 (0.82)	3.98 (0.66)	3.70 (0.94)	125.11	0.000	0.669	0.000	0.000	0.000
Amusement	2.17 (0.70)	3.35 (0.84)	4.02 (0.77)	3.48 (0.93)	94.75	0.000	0.604	0.000	0.000	0.000
Attractiveness	3.02 (1.10)	2.83 (0.83)	2.40 (0.95)	2.90 (1.14)	1.43	0.235	0.023	0.882	0.008	0.009
**(Range 0–8)**										
Happiness	1.92 (1.33)	3.83 (1.59)	4.87 (1.44)	4.13 (1.51)	101.58	0.000	0.621	0.000	0.000	0.000
Sadness	3.51 (2.23)	1.86 (1.64)	1.19 (1.37)	1.83 (1.68)	40.53	0.000	0.409	0.000	0.000	0.001
Surprise	1.35 (1.03)	2.29 (1.47)	3.29 (1.74)	3.00 (1.58)	83.93	0.000	0.575	0.000	0.000	0.000

Multiple comparisons a posteriori using Bonferroni test: Theme (A), Variation 1 (B) and Variation 2 (C). Critical value of p < 0.050.

An increase in the values of emotions “Happiness” and “Surprise,” as well as in descriptive scales “Tension,” “Expressiveness,” and “Amusement” occurs in the use of rhythmic variations 1, 2, and 3 in comparison with the main theme. The gotten order of the scores is described next for these parameters. The use of whole and half notes (the theme) is punctuated with lower values for “Happiness,” “Surprise,” “Tension,” “Expressiveness,” and “Amusement,” followed by the use of eighth notes (variation 1). Then we find higher scores for the use of short syncopations, dots contained in a pulse and triplets of eighth notes (that is, variation 3). A syncopation is a deliberate upsetting of the meter or pulse of a composition by means of a temporary shifting of the accent to a weak beat or an off-beat. And, a doted note is a note that has a dot placed to the right of the note head, indicating that the duration of the note should be increased by half again its original duration.

Finally, you may observe that the use of sixteenth notes and their combinations present in variation 2 are valued with the highest scores in these parameters. These are “Happiness,” “Surprise,” “Tension,” “Expressiveness” and “Amusement.” The same order of punctuation for the theme and three variations is followed for “Sadness” emotion but in reverse order. The use of sixteenth notes (variation 2) is perceived as the less sad in this emotion, followed by variation 3, variation 1 and theme. Again, “Attractiveness” offers non-significant values, so, we will not consider the scores gotten.

**Figure 5:**
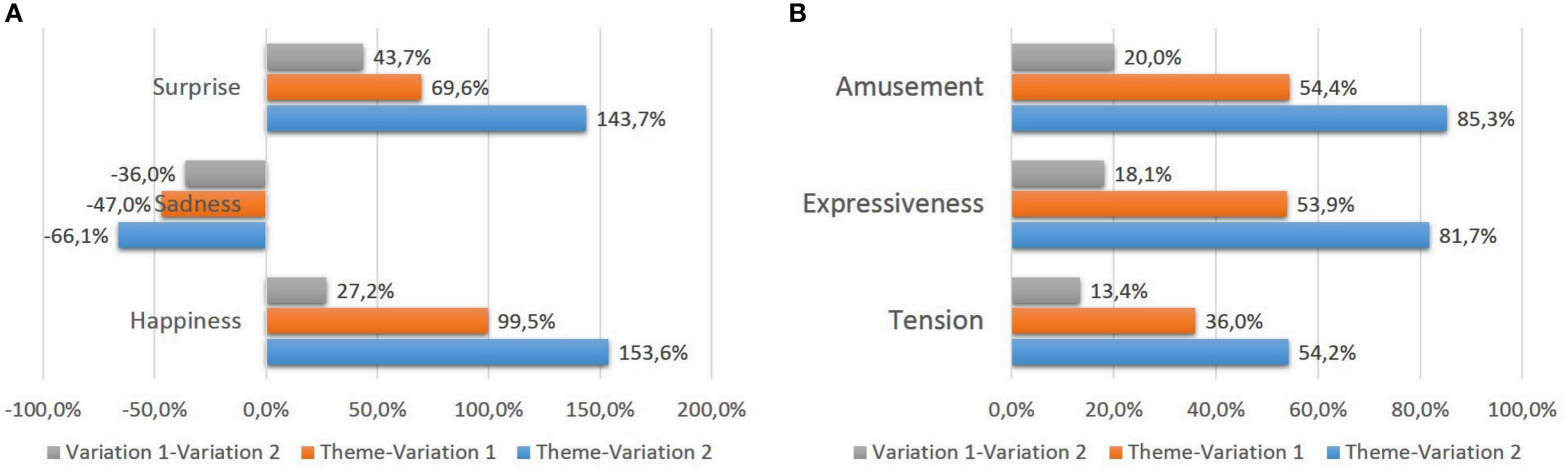
**Growth tendencies for experiment 2**. **(A)** Tendencies in basic emotions. **(B)** Tendencies in descriptive scales.

In all the emotions and descriptive words that suffer an increase/decrease in their values due to the proposed variations from the main theme (“Happiness,” “Surprise” and “Sadness”; “Tension,” “Expressiveness” and “Amusement”), it shows that growth occurs significantly in all of them, albeit with different growth percentages (see Figure 5). When passing from the theme to variation 2, the two highest increases occur for the values of “Happiness” (increased by 153.6% when playing sixteenth compared to the use of whole and half notes) and “Surprise” (increased by 143.7%). Other emotional judgments increase in a high percentage above 50% (“Amusement,” “Expressiveness,” and “Tension”). In the case of “Surprise” there is a decrease in a value of 66.1%. Again, this emotion is opposed to the feeling of “Happiness.”

Moreover, it is interesting to compare the results expressed when passing from whole and half notes (theme) to eighth notes (variation 1) and from eighth notes to sixteenth notes (variation 2). This is why, it is checked if there is proportionality in the emotional perception results, as occurs in the configuration of the proper rhythms. Thus, for those emotions in which an increase in their scores does occur, it is studied whether they follow a principle of binary multiplication results, coinciding with the structure of the rhythms (eighth notes are the result of a binary regular division of the half notes, like sixteenth notes result from a binary regular division of the eighth notes). In all cases, you can observe that there is a stronger effect in the increase/decrease of the scores when passing from theme to variation 1 vs. variation 1 to variation 2. This is especially pronounced for emotion “Happiness,” followed by “Surprise,” whilst “Sadness” experiences a lower difference. All the three remaining descriptive words also experience a very high difference in increase.

## 5. Discussion

### 5.1. Effects of tempo and rhythmic unit on emotional perception

**Figure 6:**
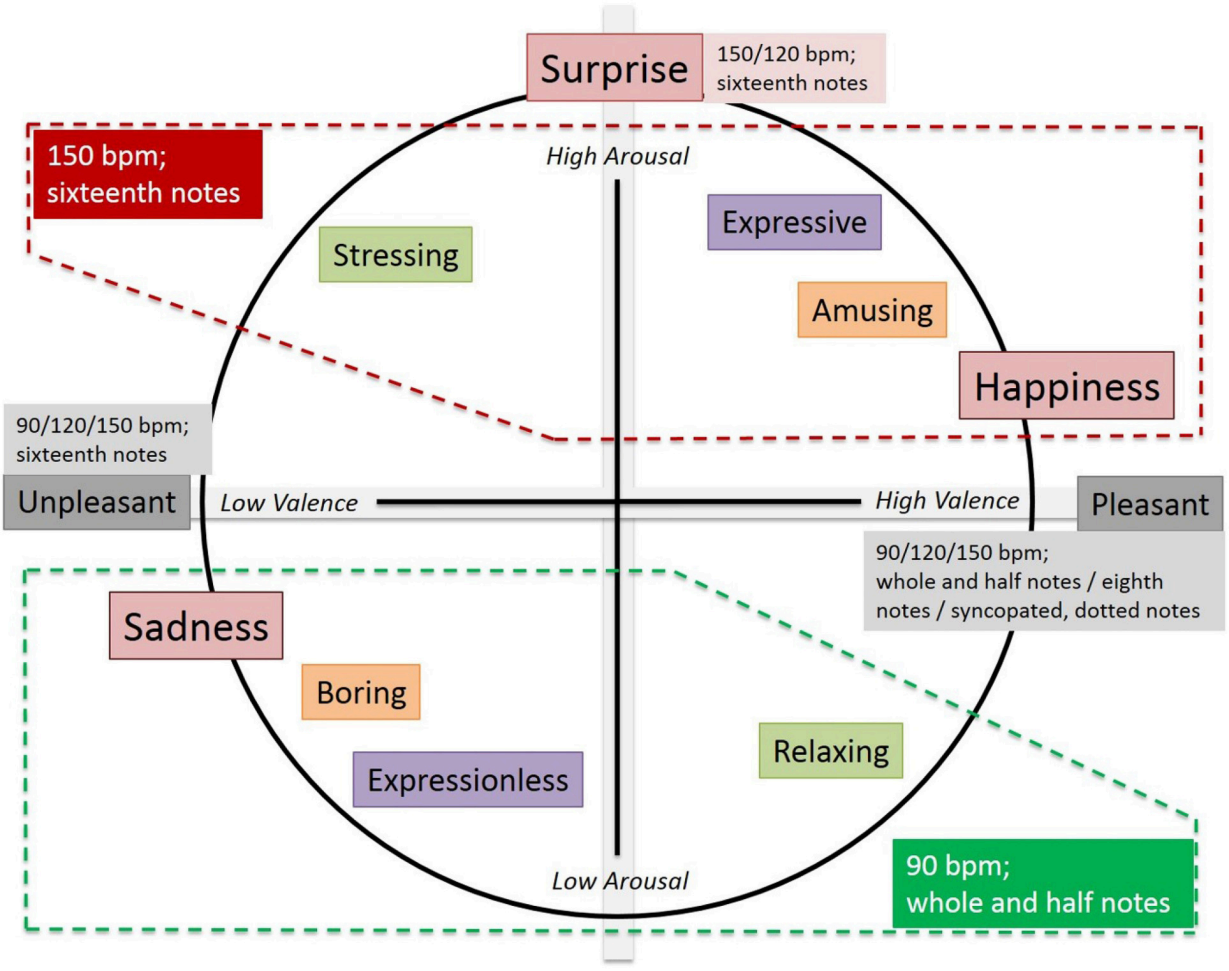
**Annotated circumplex emotion model**.

In order to discuss on the individual influence or effect of tempo and rhythmic unit on emotion regulation, we have used the well-known circumplex model of affect (Russell, 1980). This model suggests that emotions are distributed in a two-dimensional circular space, containing arousal and valence dimensions. Arousal represents the vertical axis and valence represents the horizontal axis, while the center of the circle represents a neutral valence and a medium level of arousal. In this model, emotional states can be represented at any level of valence and arousal, or at a neutral level of one or both of these factors. Circumplex models have been used most commonly to test stimuli of emotion words, emotional facial expressions, and affective states. As you can observe in see Figure 6, we have annotated the standard circumplex emotion model with the three emotions studied, that is, “Happiness,” “Sadness,” and “Surprise,” as well as the three meaningful opposed semantic scales related to “Amusement,” “Expressiveness,” “Tension,” and “Attractiveness.” We have also annotated the tempo and rhythmic unit values that were rated with higher scores for emotions and semantic scales.

The figure offers a symmetric appearance. In the middle value of arousal there is “Attractiveness,” showing that there is no influence of tempo and/or rhythmic unit. All combinations of tempo (90, 120, and 150 bpm) provide similar outputs for pleasantness. In relation to rhythmic unit, (*Pleasant* seems to be achieved with sixteenth notes and *Unpleasant*) with all the rest of possibilities. But, as seen in the Results sections, there is statistically not enough significance. At the top of Figure 6 the emotion “Surprise” appears quite isolated from the rest of emotions. Again, there are no clear outperforming tempo values (120 and 150 bmp) or rhythmic unit value (sixteenth notes) in accordance with the statistical results obtained before. Nevertheless, the most important result that can be seen in the figure is related to opposing emotions “Happiness” and “Sadness,” and couples of feeling words *Amusing* vs. *Boring, Expressive* vs. *Expressionless*, and *Stressing* vs. (*Relaxing*. All the first terms are related to a relatively high arousal whilst the second ones are in the low arousal level. Moreover, the first terms get their maximum score with a tempo of 150 bpm and a rhythmic unit of sixteenth notes. On the contrary, the second terms perform best with a 90 bpm tempo, and whole and half notes as rhythmic unit.

The results obtained in relation to tempo and its influence on emotional perception are clearly in line with previous research works. Indeed, the suggestion that emotion conveyed by music is determined by mode (major-minor) and tempo (fast-slow) was examined using the same set of equitone melodies in two experiments (Gagnon and Peretz, 2003). The results confirm that both mode and tempo, with tempo being more prominent, determine the “happy-sad” judgments in isolation. Thereby, tempo and mode provide essential factors to determine whether music sounds sad or happy. Slow tempo and minor mode are associated with sadness whereas music played with fast tempo and composed in major mode is commonly considered happy (for a review, see Juslin and Laukka, 2003). Moreover, tempo variation has consistently been associated with differential emotional responses to music (Dalla Bella et al., 2001; Gagnon and Peretz, 2003). For example, Rigg (1940) examined college students emotional responses to each of five different phrases presented at six tempos ranging from 60 to 160 quarter notes or beats per minute (bpm) varying in steps of 20 bpm. Across the five manipulated phrases, as tempo increased, more students were likely to describe the phrases as happy than sad. In contrast, more recent research has suggested that changes in tempo are more closely associated with changes in arousal than emotion *per se* (Husain et al., 2002). It is conceivable that the extraction of temporal information and the following interpretation in regard to emotion has a biological foundation because tempo is regarded as a domain-general signal property. For instance, fast tempo is commonly associated with heightened arousal (Trehub et al., 2010). Another study provides a further investigation of mode and tempo (Trochidis and Bigand, 2013). It investigates the effect of three modes (major, minor, locrian) and three tempos (slow, moderate, fast) on emotional ratings and EEG responses. Beyond the effect of mode, tempo was found to modulate the emotional ratings, with faster tempo being more associated with the emotions of anger and happiness as opposed to slow tempo, which induces stronger feeling of sadness and serenity. This suggests that the tempo modulates the arousal value of the emotions. A new point of the study was to manipulate three values of tempo (slow, moderate, fast). The findings suggest that there is a linear trend between tempo and the arousing values of the emotion: the faster the tempo, the stronger the arousal value of the emotion.

On the other hand, let us highlight that, as far as we have seen, the effect of rhythmic unit has not been studied so far in musical emotion regulation. This is why, in consonance with Figure 6, one question arises. Is there a relationship between tempo and rhythmic unit that effect on emotion regulation? Some previous research can provide some hints on this issue. Firstly, a psychological investigation (Krumhansl, 2000) has deduced that temporary construction in music, turned into rhythmic patterns, not only results in complex components in music, but also complex psychological representations mainly influencing perception. Thus, the proportions of duration in music tend to simple ratios (1/2, 1/3), producing a psychological assimilation of these categories. The pulse provides a periodic base structure that allows the perception of specific temporal patterns. Thus, a framework to organize and remember the events taking place in time is provided. The following three studies conclude that the proposed relationship between tempo and rhythmic un it really exists. In the work by Desain et al. (2000), different rhythms are offered, in which the pulse (40, 60, and 90 bpm) is changed, and the bars also vary. The rhythmic patterns are perceived differently when the pulse and bars in which they are written change. Furthermore, this variation occurs, in varying ways, depending on its complexity. In another experiment (Bergeson and Trehub, 2005), different listening rhythms were offered to 7-month babies to discuss their response to rhythmic variations (some in a particular rhythmic accentuation frame, some without, and with the use of binary and ternary measure). Babies detected rhythmic changes more easily in the rhythmic context with clear accentuation (rhythm that corresponds to the measure). From the regularity of the perceived accents temporal changes in listening could be detected. Moreover, babies detected the rhythms much more clearly in binary than in ternary compass. Lastly, in Seyerlehner et al. (2007) the relationship between rhythmic patterns and tempo perceived in them is studied. Is is concluded that listeners perceive a similar tempo when they hear songs with similar rhythmic patterns. The effectiveness to discriminate each rhythmic pattern depends largely on training, similarity and capacity of the rhythmic patterns used to capture and regroup the regular rhythmic by the listener.

Clearly, in accordance with the results of the experiments, and after taking a look at Figure 6, sixteenth notes and 150 bpm are closely related, as well as 90 bpm and whole and half notes. Rhythmic unit related to syncopated notes deserves a special paragraph. Although syncopated/dotted notes have not been determinant in the experiments performed in the present study, some of the results gotten here are also in line with musical emotion research. Indeed, it is well-known that syncopated patterns are perceived as more fun and upbeat than non syncopated. Moreover, a syncopated pattern is always perceived as more complex and exciting than a non-syncopated one. This difference increases when a non-syncopated pattern is followed by a syncopated one (Keller and Schubert, 2011).

### 5.2. Toward musical emotion regulation through note value

Obviously, the results offered in Figure 6 are not sufficient to draw conclusions on how tempo and rhythmic unit (be it individually or combined) can be used to regulate emotions. It is even not possible to provide sufficient evidence on how to move from a negative to a positive mood by playing music with different tempo and/or rhythmic unit. Indeed, in the previous discussion only arousal has been introduced. But the questions is also “What happens with valence?”

If we only rely on 150 bpm and sixteenth notes vs. 90 bpm and whole and half notes, we clearly attain a higher or lower arousal level. But we do not know to what extent a person feels “Happiness” at the same time that he/she finds music to be *Stressing, Expressive* and *Amusing*. “Happiness” is for sure a positive emotion, and *Expressive* and *Amusing* are good friends for the emotion. But, is something *Stressing* also positive? At the opposite side, we have the just the contrary. The emotion felt is “Sadness” and the music is rated as *Boring, Expressionless* and *Relaxing*. “Sadness,” *Boring* and *Expressionless* perfectly fit under a negative emotional impression, but *Relaxing* is a positive feeling.

**Figure 7:**
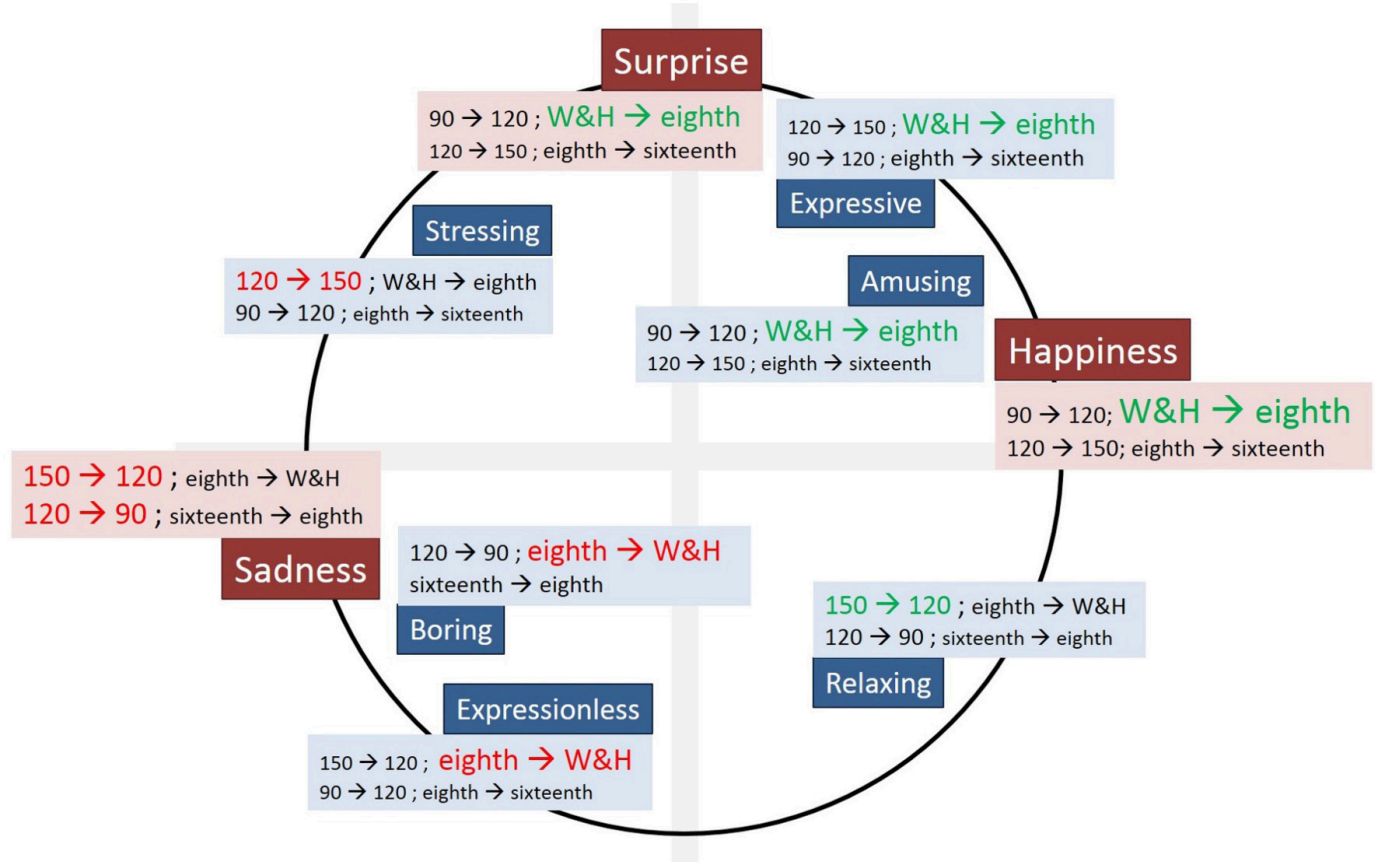
**Annotated circumplex emotion model**.

So, there must be a solution that makes things function correctly. This is why, we have prepared another figure that enables to offer a better understanding of the combined smart use of tempo and rhythmic unit to regulate emotions. Figure 7 shows, for each emotion and descriptive word, the influence of tempo (both partial increment from 90 to 120 or decrement from 120 to 90 bpm, and increment from 120 to 150 bpm or decrement from 150 to 120 bpm) combined with the most influential change in rhythmic unit for that specific feeling (in positive: white and half notes (W&H) to eighth notes (eighth) and eighth notes to sixteenth notes (sixteenth); in negative: sixteenth to eighth and eighth to W&H). The size of the font determines the importance of the influence, that is, the biggest the font, the greatest the influence. We have also used green and red color to depict the most influential value to move toward a negative (red) and negative (green) emotional state.

For instance, to reach “Happiness” Figure 7 suggests to use a tempo of 120 bpm (passing from 90 to 120 bpm (90 → 120), if necessary) and clearly establishes that you have to move rhythmic unit from whole and notes to eighth notes (W&H → eighth). Notice that this combination includes the positive feelings of *Expressive* and *Amusing*. This combination of parameters of note value do not get a feeling of *Stressing*, as this is achieved only if you pass from 120 to 150 bpm. And, the opposite problem also gets a correct solution in Figure 7. Obviously new experiments have to be carried out to demonstrate the validity of this approach.

## 6. Conclusions

This article has described the first steps in the use of music to regulate affect in a running project denominated “Improvement of the Elderly Quality of Life and Care through Smart Emotion Regulation.” The objective of the project is to find solutions for improving the quality of life and care of aging adults living at home by using emotion elicitation techniques. This paper has focused on emotion regulation through some musical parameters.

The proposal has studied the participants' changes in emotional states through listening different auditions. The present research has focused on the musical cue of note value through two basic components of the parameter note value, namely, tempo and rhythmic unit to detect the individual preferences of the listeners. The two experiments carried out have been discussed in detail to provide an acceptable manner of using both parameters to be able to understand how to move from negative to positive emotional states (and vice versa), which is one key of our current project.

The results obtained in relation to tempo and its influence on emotional perception are in line with previous research works. The results confirm that tempo clearly determines whether music sounds sad or happy. Moreover, Stressing, Expressive and Amusing are the words gotten from a high tempo, whilst the opposite terms Relaxing, Expressionless and Boring are obtained for a low tempo. This suggests that the tempo modulates the arousal value of the emotions. On the other hand, the effect of rhythmic unit, which has not been studied in musical emotion regulation, has shown significant outcomes in the same direction than tempo. Indeed, sixteenth notes and 150 bpm are closely related, as well as 90 bpm and whole and half notes.

Lastly, the paper has established a basis for future study of the combined effect of both parameters so to provide a finer tuning of the emotion regulation capabilities of the proposed system.

## Author contributions

All authors listed, have made substantial, direct and intellectual contribution to the work, and approved it for publication.

## Funding

This work was partially supported by Spanish Ministerio de Economía y Competitividad/FEDER under TIN2013-47074-C2-1-R, TIN2015-72931-EXP and DPI2016-80894-R grants.

### Conflict of interest statement

The authors declare that the research was conducted in the absence of any commercial or financial relationships that could be construed as a potential conflict of interest.
